# Field evaluation of selected cassava genotypes for cassava brown streak disease based on symptom expression and virus load

**DOI:** 10.1186/s12985-014-0216-x

**Published:** 2014-12-20

**Authors:** Tadeo Kaweesi, Robert Kawuki, Vincent Kyaligonza, Yona Baguma, Geoffrey Tusiime, Morag E Ferguson

**Affiliations:** National Crops Resources Research Institute, Root Crop Program, Namulonge, Uganda; Makerere University, College of Agricultural and Environmental Sciences, Kampala, Uganda; International Institute of Tropical Agriculture (IITA), c/o ILRI, P.O Box 30709, Nairobi, 00100 Kenya

**Keywords:** Cassava, Cassava brown streak viruses, Resistance mechanism, Virus accumulation

## Abstract

**Background:**

Production of cassava (*Manihot esculenta* Crantz), a food security crop in sub-Saharan Africa, is threatened by the spread of cassava brown streak disease (CBSD) which manifests in part as a corky necrosis in the storage root. It is caused by either of two virus species, *Cassava brown streak virus* (CBSV) and *Ugandan cassava brown streak virus* (UCBSV), resulting in up to 100% yield loss in susceptible varieties.

**Methods:**

This study characterized the response of 11 cassava varieties according to CBSD symptom expression and relative CBSV and UCBSV load in a field trial in Uganda. Relative viral load was measured using quantitative RT-PCR using COX as an internal housekeeping gene.

**Results:**

A complex situation was revealed with indications of different resistance mechanisms that restrict virus accumulation and symptom expression. Four response categories were defined. Symptom expression was not always positively correlated with virus load. Substantially different levels of the virus species were found in many genotypes suggesting either resistance to one virus species or the other, or some form of interaction, antagonism or competition between virus species.

**Conclusions:**

A substantial amount of research still needs to be undertaken to fully understand the mechanism and genetic bases of resistance. This information will be useful in informing breeding strategies and restricting virus spread.

**Electronic supplementary material:**

The online version of this article (doi:10.1186/s12985-014-0216-x) contains supplementary material, which is available to authorized users.

## Background

Cassava brown streak disease (CBSD) has been identified among the seven most serious threats to world food security [1]. Leaf symptoms include blotchy yellow chlorosis or feathery necrosis, often associated with minor veins, which can appear within the first few months after planting of infected cuttings and persist in mature leaves. Brown, round or elongate streak-like lesions can occur on the young green portion of infected stems, but the main economic loss is caused by dry, brown necrotic lesions in the storage tissues of the tuberous roots of infected susceptible plants [2–4]. Root constrictions are also sometimes observed as well as brown/black lesions on green fruits, and necrotic lesions in leaf scars. In severe infections these lesions develop to kill the dormant axilliary buds leading to a general shrinkage of the node and death of the intermodal tissue, so that the branch dies from the tip to cause ‘dieback’ [5]. Secondary losses occur as a consequence of early harvesting, which farmers use as a strategy to avoid root necrosis [6].

CBSD is caused by at least two distinct virus species; *Cassava brown streak virus* (CBSV), and *Uganda cassava brown streak virus* (UCBSV), both picorna-like (+) ssRNA viruses from the genus *Ipomovirus*, family *Potyviridae* [7,8]. These viruses spread along with the infected vegetative planting material and are also transmitted in a semi-persistent manner by whitefly, *Bemisia tabaci* [9]. For the first approximately 70 years that CBSD was recognized [2] it occurred at relatively low levels in coastal East Africa, from Mozambique in the south to north-eastern Kenya in the north, and inland to the shores of Lake Malawi [3,5]. In the early 2000s, however, new outbreaks were reported from south-central Uganda [10], western Kenya (H.M. Obiero, personal communication) and north-western Tanzania [11]. More recently CBSD has been reported from Burundi [12], Rwanda [13] and the Democratic Republic of Congo [14], indicating a possible spread to West Africa. The spread of CBSVs has been fuelled by so-called ‘super-abundant’ whiteflies, *Bemisia tabaci* [4,15].

Breeding for resistance to cassava mosaic disease (CMD) and CBSD was initiated in 1937 in Amani, Tanzania and due to insufficient levels of resistance in cultivated cassava, a strategy to incorporate resistance from wild species, particularly from *M. glaziovii* and *M. melanobasis* (now regarded as *M. esculenta subsp. flabellifolia* [16]), through inter-specific hybridization and backcrossing was adopted [17,18]. Several of these inter-specific hybrids have been incorporated into the farming systems in the region and are now considered as ‘farmer varieties’ or landraces. One of the most resistant of these is known as ‘Kaleso’ in Kenya and ‘Namikonga’ in Tanzania [5,19]. Today these form an important genepool for CBSD resistance breeding and some of the genotypes used in this study are derived from the Amani breeding program.

Severity of CBSD symptom expression varies considerably with cassava varieties and with the environment [5,18]. Some varieties show severe shoot and root symptoms while others show either marked leaf symptoms and mild root necrosis or *visa versa*, as well as combinations of milder versions of both leaf and root symptoms [5,20]. Recent evidence from a graft-innoculated cassava glasshouse study showed that ‘resistant’ and ‘tolerant’ varieties, with mild symptoms, restrict virus accumulation in the plant and support lower virus titres than susceptible genotypes [21]. This supports the findings of others [22] and suggests that ‘tolerant’ varieties possess molecular resistance mechanisms that impair the replication of CBSVs. Although different levels of resistance/tolerance are recognized, no immunity has been observed. In this study genotypes were systematically evaluated under field conditions to quantify their response to virus infection and determine the relationship between relative virus load, symptom type and severity.

## Results

### CBSD shoot symptom severity and incidence

**Figure 1:**
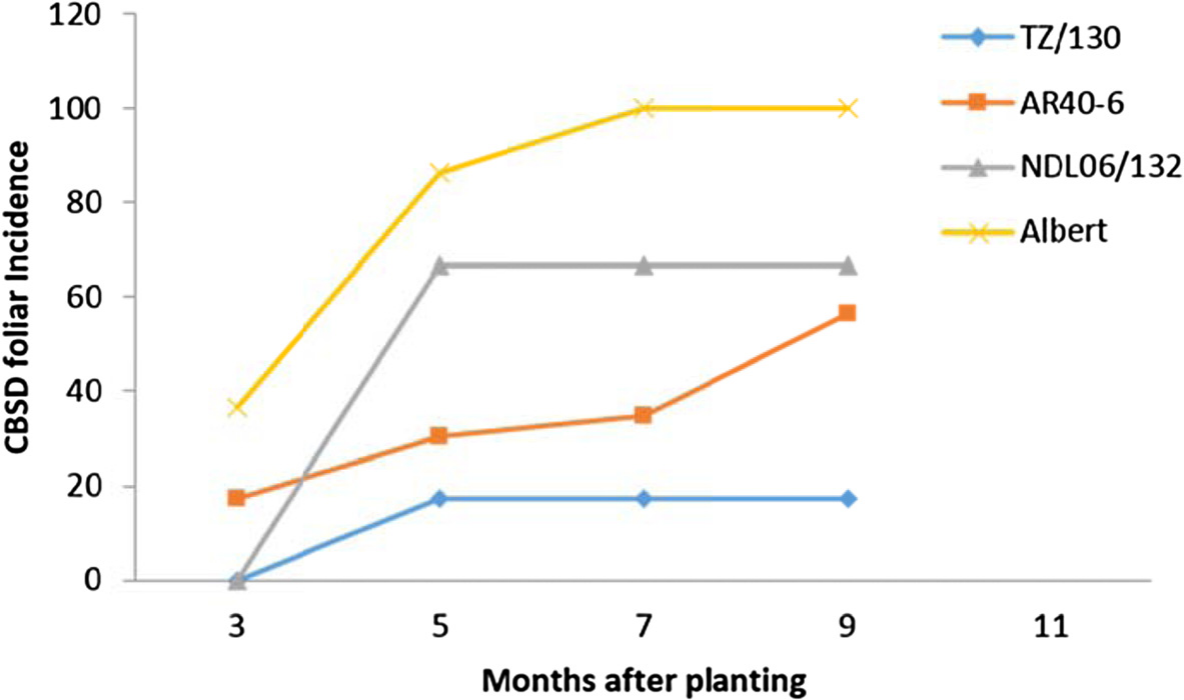
**CBSD shoot incidence in selected genotypes with time.**

Genotypes NASE 14, NASE 1, Kiroba and NASE 19 did not show shoot symptoms during the duration of the experiment (Table 1). Of those genotypes that showed symptoms, Namikonga and TZ/130 had the lowest mean incidence of 9% and 17% and mean shoot severity of 1.09 and 1.17 respectively, while known CBSD susceptible varieties, Albert and TME 204, showed severe shoot symptoms with mean shoot severity of 3 and 4.07 respectively and mean incidence of 100% (Table 1). Shoot symptoms that were observed as early as 3MAP persisted up to the time of harvest (Figure 1). Maximum CBSD shoot symptom incidence was observed at 5MAP in genotypes TZ/130 and NDL06/132, while in other genotypes such as Albert and AR40-6, the disease incidence continued to rise after 5MAP (Figure 1). Higher abscission was noted among the lower leaves on which symptoms predominate.

**Table 1:** **Shoot and root CBSD symptom incidence and severity, coefficient of determination (r** ^**2**^ **) between virus load and mean shoot symptom expression, and harvest index**

**Genotype**	**Shoot incidence % (9MAP)**	**Shoot symptom severity (9MAP)**				**Root necrosis incidence %**	**Root necrosis severity**				**Coefficient of determination (r** ^**2**^ **) between virus titre and mean shoot symptoms at 3,5,7,9 and 11MAP**		**Harvest index**
		**Mean**	SD*	**Min**	**Max**		**Mean**	SD*	**Min**	**Max**	**UCBSV**	**CBSV**	
**NASE 14**	0	1.00	0.00	1	1	31.7	1.35	0.88	1	5	-	-	0.37
**Kiroba**	0	1.00	0.00	1	1	14.3	1.07	0.12	1	3	-	-	0.36
**NASE 1**	0	1.00	0.00	1	1	18.0	1.05	0.09	1	2	-	-	0.35
**NASE 19**	0	1.00	0.00	1	1	67.0	2.15	1.52	1	5	-	-	0.26
**Namikonga**	9	1.09	0.30	1	2	10.0	1.03	0.04	1	2	0.37	0.67	0.15
**TZ/130**	17	1.17	0.39	1	2	38.3	1.20	0.67	1	4	0.17	0.33	0.44
**AR40-6**	52	1.61	0.58	1	3	30.3	1.09	0.28	1	3	0.16	0.97	0.49
**Kibaha**	75	2.25	0.89	1	3	67.4	2.75	1.00	3	5	0.93	0.35	0.37
**NDL06/132**	67	2.30	1.53	1	4	39.7	1.53	0.40	2	3	0.92	0.67	0.49
**Albert**	100	3.00	0.00	3	3	66.3	2.54	1.23	1	5	0.96	0.52	0.29
**TME 204**	100	4.07	0.55	3	5	100	4.78	0.39	4	5	0.84	0.53	0.16

*SD – Standard deviation.

### CBSD root symptom severity and incidence

One of six Namikonga plants showed the mildest of symptoms (Class 2) (Tables 1 and 2). It had the highest proportion of plants with no root necrosis (83.3%), followed by NASE 1 and AR40-6 with 73.3% and 63.6% respectively (Table 2). All plants in NDL06/132, Kibaha and TME 204 showed at least one root with root necrosis. Seven Kibaha and 10 TME 204 plants showed symptoms with a maximum score of 5. Namikonga and NASE 1 had a maximum root necrosis severity score of 2, while AR40-6, Kiroba and NDL06/132 scored 3 and TME 204, Albert, Kibaha and NASE 14 all scored 5 (Table 1).

**Table 2:** **Number of plants per variety with plant root mean disease incidence in a given range**

**Genotypes**	**Number of plants showing per plant mean root disease incidence**					**Total number of plants assessed per genotype**	**Total number of roots assessed per genotype**	**% symptomless plants**
	**0%**	**1-5%**	**6-25%**	**26-75%**	**>75%**			
NASE 14	7	5	1	1	1	15	114	46.7
Kiroba	3	3	1	0	0	7	54	42.9
NASE 1	11	3	1	0	0	15	72	73.3
NASE 19	2	2	0	1	4	9	82	22.2
Namikonga	5	1	0	0	0	6	37	83.3
TZ/130	11	4	3	1	1	20	164	55
AR40-6	14	6	2	0	0	20	164	63.6
Kibaha	0	0	2	2	3	7	60	0
NDL06/132	0	2	2	0	2	6	73	0
Albert	1	3	5	2	9	20	93	5
TME 204	0	0	0	0	10	10	34	0

Interestingly 15 of NASE 14 plants were asymptomatic for both shoot and root symptoms, five showed mild symptoms and two showed very high severity (4 or 5) and incidence (90–100) on roots. This was coupled with reduction in growth and in some cases dieback.

### Detection and quantification of UCBSV and CBSV

**Figure 2:**
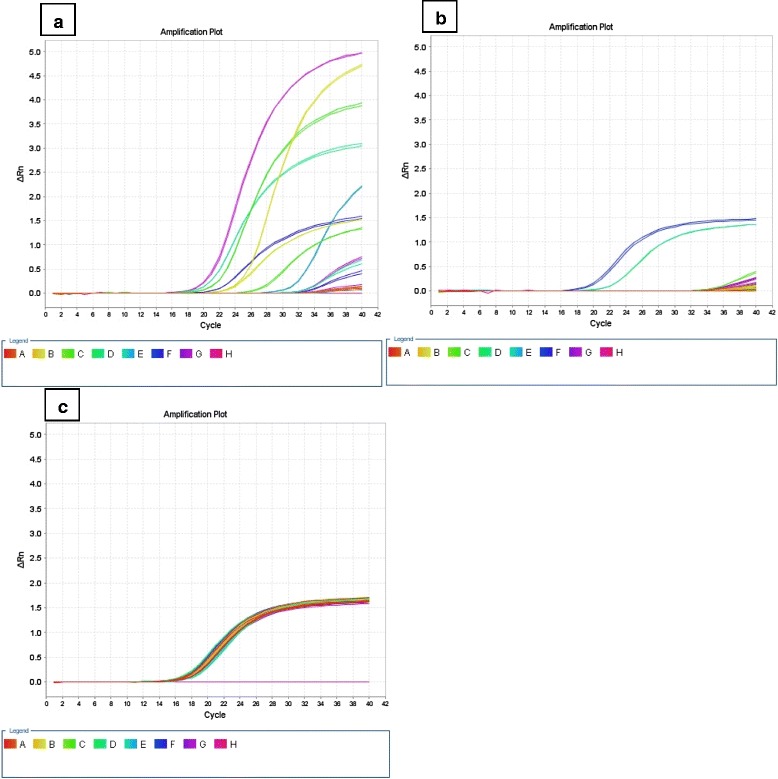
**Amplification plots at 11MAP for (a) CBSV, (b) UCBSV and (c) COX, the housekeeping gene.** From the amplification plot, the Ct values for CBSV in most genotypes were detected earlier **(a)** and showed exponential increase as compared to those of UCBSV **(b)** in the same genotypes. Legend represents different genotypes: NASE 14 (A), AR40-6 (B), Kibaha (C), NDL06/132, Kiroba (E), Albert (F) and non-template control (G).

Both UCBSV and CBSV were detected in all varieties at some stage during the growing season. None of the varieties were immune. Amplification plots are shown in Figure 2 at 11MAP for CBSV, USBSV and COX. CBSV was detected at 3MAP in all varieties except Kiroba, Kibaha, Namikonga and NASE19, which showed infection at 5MAP (Table 3). Similarly UCBSV was detected in all varieties except Kiroba, NASE 1 and Kibaha. However by 5MAP, UCBSV could be detected in all varieties except NASE1 which started showing infection by 9MAP (Table 3). Interestingly, after detection at 5 and 7MAP, UCBSV was undetectable in Kiroba 9 and 11MAP. Absolute C_t_ values of both UCBSV and CBSV observed in the selected genotypes at 3,5,7,9 and 11 MAP are presented in Additional file 1: Tables S1 and S2.

**Table 3:** **Detection (presence/absence) of CBSV and UCBSV in the selected genotypes during the course of the infection**

**Genotype**	**3MAP**		**5MAP**		**7MAP**		**9MAP**		**11MAP**	
	**UCBSV**	**CBSV**	**UCBSV**	**CBSV**	**UCBSV**	**CBSV**	**UCBSV**	**CBSV**	**UCBSV**	**CBSV**
**NASE 14**	**+**	**+**	**+**	**+**	**+**	**+**	**+**	**+**	**+**	**+**
**Kiroba**	**_**	**_**	**+**	**+**	**+**	**+**	**_**	**+**	**_**	**+**
**NASE 19**	**+**	**_**	**+**	**+**	**+**	**+**	**+**	**+**	**+**	**+**
**Namikonga**	**+**	**_**	**+**	**+**	**+**	**+**	**+**	**+**	**+**	**+**
**TZ/130**	**+**	**+**	**+**	**+**	**+**	**+**	**+**	**+**	**+**	**+**
**NASE 1**	**_**	**+**	**_**	**+**	**_**	**+**	**+**	**+**	**+**	**+**
**Kibaha**	**_**	**_**	**+**	**+**	**+**	**+**	**+**	**+**	**+**	**+**
**Albert**	**+**	**+**	**+**	**+**	**+**	**+**	**+**	**+**	**+**	**+**
**AR40-6**	**+**	**+**	**+**	**+**	**+**	**+**	**+**	**+**	**+**	**+**
**NDL06/132**	**+**	**+**	**+**	**+**	**+**	**+**	**+**	**+**	**+**	**+**
**TME 204**	**+**	**+**	**+**	**+**	**+**	**+**	**+**	**+**	**+**	**+**

+ pooled sample tested positive for the virus; − pooled sample tested negative for the virus.

Though both virus species were detected in all the genotypes, the viral load differed among genotypes. At the final sampling time-point (11MAP), genotype NASE 14, had the least relative viral load for both UCBSV and CBSV i.e. 1.16 and 0.00071 folds (ΔΔCt), respectively (Table 4). As the fold change at 5,7,9 and 11 MAP is calculated relative to the ΔCt value at 3MAP, and since CBSV was detected at 3 MAP (Ct values of the technical reps were 21.32 and 23.86 (Additional file 1: Table S2), the value of 0.00071 indicates that the virus was present but there was little if any change in virus load relative to 3MAP, taking into consideration the small variations in Ct values of the internal controls. Other genotypes with comparatively low virus titre for UCBSV included Kiroba (0.7), AR40-6 (0.026), TZ/130 (1.72), Namikonga (9.25) and NASE 19 (16.11). Genotype NDL06/132 had the highest relative UCBSV viral load (353169.2). For CBSV, Kiroba, NASE 19 and Namikonga also had comparatively low relative viral loads of 30.1, 165.4 and 199.5 folds respectively. Genotype NDL06/132 had the highest virus titre of 294927.33 folds (Table 4).

**Table 4:** **Accumulation of UCBSV and CBSV in selected genotypes with time (fold change relative to 3MAP, ΔΔCt)**

**Genotypes**	**5 MAP**	**7 MAP**	**9MAP**	**11 MAP**
**UCBSV**				
Namikonga	1.67	3.81	1.87	9.25
NASE 1	1.18	1.39	1.75	133.4
AR40-6	8.88	63.12	588.13	0.026
Kiroba	24.59	76.64	36.76	0.7
Tz/130	0.49	1.96	1.09	1.72
NASE 14	1.77	58.48	2.08	1.16
NASE 19	2.00	5.54	6.41	16.11
NDL06/132	22.94	48.17	2836.7	353169.2
Albert	3.66	6.19	20738.2	220435.95
Kibaha	32.45	105.42	407.31	5634.21
TME 204	4039.61	279018.26	912838.43	2039805.3
**CBSV**				
Namikonga	7804.01	153725.82	568.1	199.5
NASE 1	205.07	606437.70	15608.02	133826.1
AR40-6	9.45	95.01	224.41	294927.33
Kiroba	53.44	709.18	16270.8	30.1
Tz/130	76331.98	499456.67	236257.4	143431.3
NASE 14	6.25	86.22	0.008	0.00071
NASE 19	129.79	552.56	1287.18	165.42
NDL06/132	38165.99	1503611.1	294927.33	297978.71
Albert	32995.91	20425	110217.9	148489.36
Kibaha	1296.13	11113.30	426442.37	2836.44
TME 204	82952.6	945029.61	102837.01	318293.9

In most cases the relative concentration of CBSV was significantly higher than that of UCBSV; for example the CBSV concentration in TZ/130 and AR40-6 were 143431.3 and 294927.33 folds respectively, compared to 1.72 and 0.026 folds respectively for UCBSV. However, it is noted that genotypes Kibaha and NDL06/132 had higher relative virus loads for UCBSV than CBSV, although in these cases titres for both viruses were high (Table 4).

**Figure 3:**
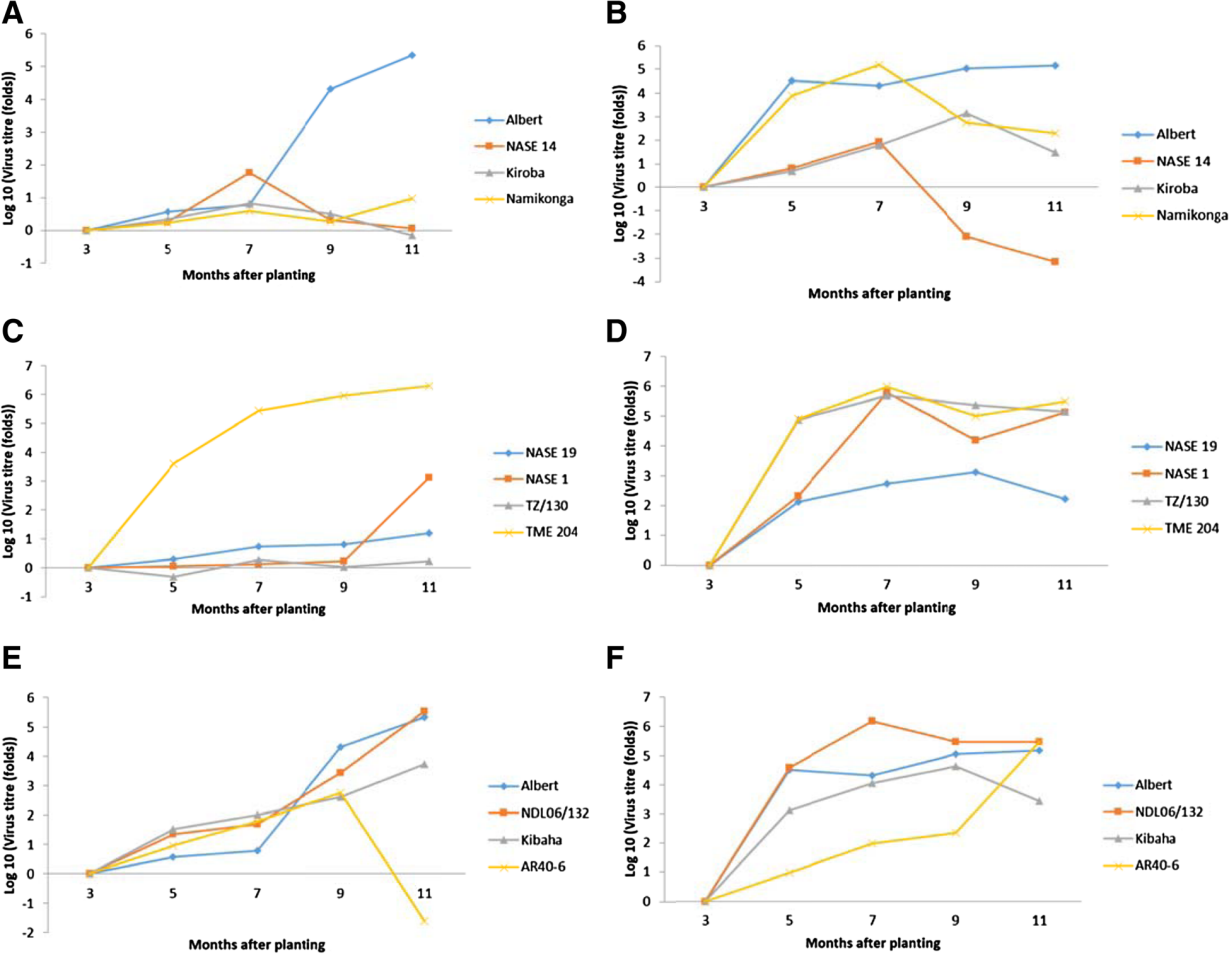
**Accumulation of both UCBSV (A, C and E) and CBSV (B, D and F) with time.**

Table 4 and Figure 3 show the progression of relative virus titre for CBSV and UCBSV from 5, 7 and 9 to 11MAP. All genotypes showed an increase in UCBSV titre between 3 – 7MAP, with the titre in the susceptible checks, Albert and TME 204 increasing dramatically at 9MAP, and continued to increase at a slower rate at 11MAP. In addition the concentration of UCBSV in NDL06/132, previously thought to be tolerant to CBSD increased substantially after 7MAP. Relative titres of UCBSV also increased in Kibaha although at much lower levels. After 7MAP the relative virus load of NASE 1 and NASE 19 also increased, but at much lower levels (132 fold and 10.57 folds respectively). UCBSV titre in NASE 14 and Kiroba continued to drop to 11MAP, but that in Namikonga rose slightly from 7 to 11MAP. In fact UCBSV could not be detected in Kiroba from 9 to 11 MAP. TZ/130 maintained a steady low virus load from 7 to 11MAP.

In general virus loads were much higher for CBSV than UCBSV. For CBSV, virus load rose in all genotypes, except Albert, up to 7 MAP (Table 4 and Figure 3). This was however at different levels and five different profiles were observed. CBSV loads were low at 5 MAP in Kiroba and NASE 14 and were also low at 11 MAP, however levels in NASE 14 remained low throughout whereas there was a peak in levels at 9 MAP (16,270) in Kiroba. Here, the consistently low levels of virus are termed CBSV Profile 1. In Namikonga virus load rose to quite high levels (153,725) at 7 MAP but then fell dramatically to 11MAP (199). A similar profile was observed in NASE 1, however the virus did not drop to such low levels (133,826). A drop in virus load at 7MAP is termed CBSV Profile 2. In NASE 19 and Kibaha CBSV levels rose to 9 MAP, then dropped to 11MAP. This is known as CBSV Profile 3. In AR40-6, levels started fairly low at 5MAP but then rose steadily to 11 MAP (294,927) (CBSV Profile 4). Levels of virus were high throughout in Albert, Tz130, NDL06/132 and TME204 (CBSV Profile 5).

### Correlation of virus load with symptom expression

**Figure 4:**
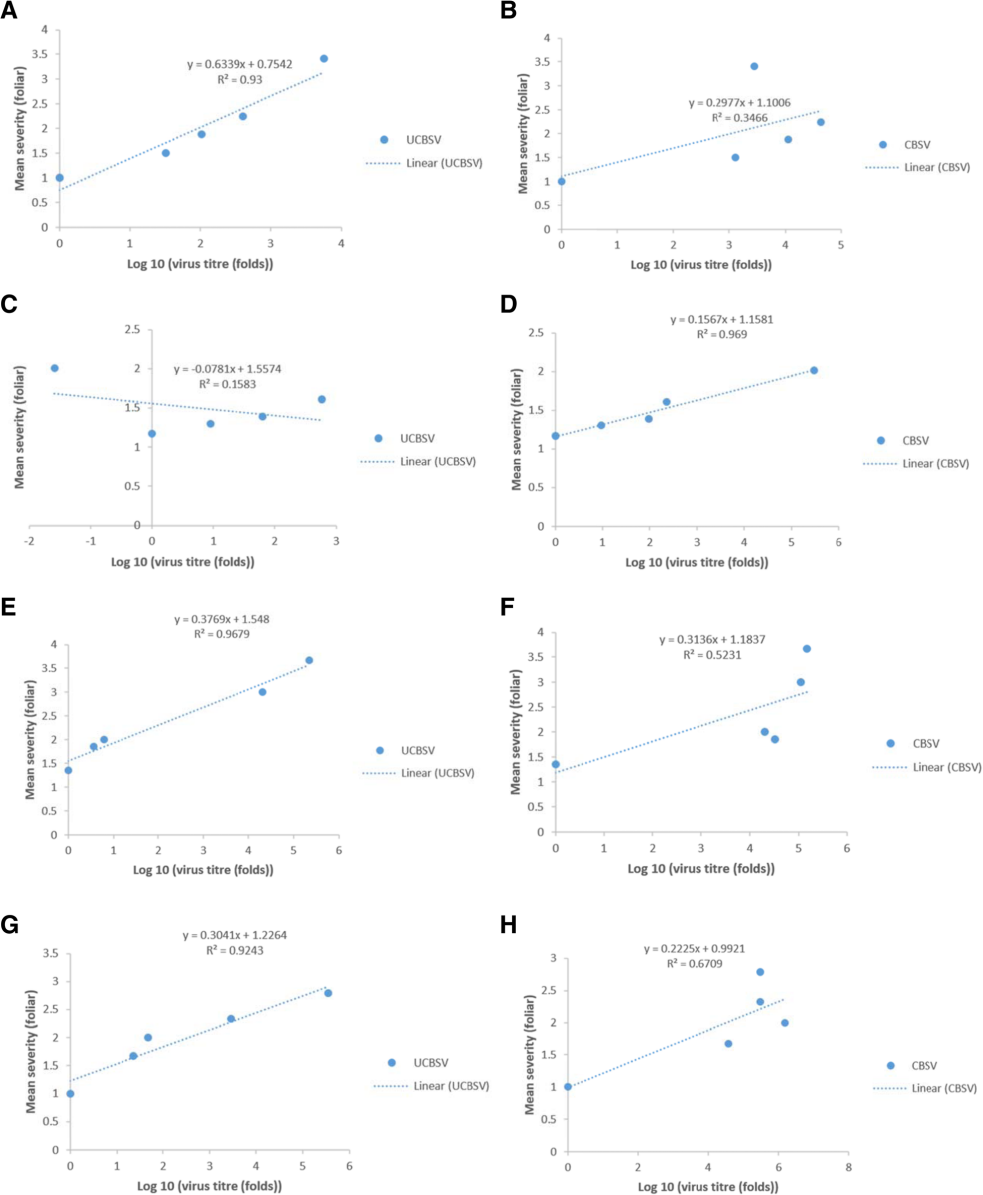
**Association between virus titre and CBSD shoot symptom development in selected cassava genotypes at 3, 5, 7, 9, and 11 MAP. A)** UCBSV in Kibaha, **B)** CBSV in Kibaha, **C)** UCBSV in AR40-6, **D)** CBSV in AR40-6, **E)** UCBSV in Albert, **F)** CBSV in Albert, **G)** UCBSV in NDL06/132 and **H)** CBSV in NDL06/132.

For varieties showing shoot symptoms the correlation of determination (r^2^) was calculated between log10 of the virus titre fold change and mean shoot symptom score at 3,5,7,9 and 11MAP (Figure 4, Table 1). A strong positive r^2^ value was observed for Kibaha (0.93), Albert (0.96) and NDL06/132 (0.92) for UCBSV and AR40-6 (0.97) for CBSV. Weak relationships and low r^2^ values were obtained for TZ/130 (0.17) and AR40-6 (0.16) for UCBSV and for TZ/130 (0.33) and Kibaha (0.35) for CBSV. In terms of root necrosis and log10 fold change in virus titre, Namikonga and to some extent Kiroba both had relatively low virus loads and root necrosis incidence and severity. NASE 14 and NASE 19 had low virus titres but high root necrosis incidence (31.7% and 67% respectively) and severity (both with maximum scores of 5). NASE 1 on the other hand had a high relative virus load of 133826 for CBSV at 11MAP but no shoot symptoms and a root necrosis incidence of 18% with a mean severity score of 1.05 and maximum of 2 (Table 1).

### Yield performance of the test genotypes at NaCRRI

Harvest index was used as an indirect assessment for fresh root yield. There was substantial variation in harvest index among the screened genotypes ranging from 0.15 – 0.49 (Table 1). Genotypes AR40–6 and NDL06/132 had the highest harvest index of 0.49, followed by TZ/130 and Kiroba with 0.46 and 0.39 respectively, while NASE 19 and Namikonga had significantly low values of harvest index of 0.26 and 0.15 respectively.

## Discussion

CBSD is a major constraint to cassava production in southern and eastern Africa, and threatens this carbohydrate staple in Central and West Africa. Continent-wide strategies are being developed to restrict the spread of the virus, including diagnostics and surveillance, prevention and control of infection using phytosanitation, and control of disease through the breeding and promotion of varieties that inhibit virus replication and/or movement [15]. Currently there is very little known about relative virus loads in field resistant/tolerant and susceptible germplasm. Even less is known about the interaction and relative competitiveness between UCBSV and CBSV in dual infections. Understanding cultivar response in relation to these aspects is important if appropriate control measures based on breeding are to be implemented, to restrict the spread of the virus. It is important that newly released varieties are either immune to the virus or restrict virus accumulation and harbor low virus load. This will reduce the source of inoculum and restrict the spread of the virus. Although a number of studies have been performed under glasshouse conditions using artificial inoculation [21–23], few field based studies have been reported under natural infection. Here we investigate symptom expression and CBSV and UCBSV relative loads over time under field conditions in 11 cassava varieties, eight of which have been classified as tolerant or resistant to CBSD in Uganda and/or Tanzania based on symptom incidence and severity in the field. It is anticipated that this type of analysis will be standardized and mainstreamed in cassava breeding.

CBSD tolerant materials were sourced from breeding programs in Tanzania (AR40-6, NDL06/132, Kiroba and Namikonga), Uganda (NASE 1, NASE 14, NASE 19 and TZ/130) and The International Institute of Tropical Agriculture, Nigeria, (TME 204). The experiment was conducted with virus-free cassava stakes over 12 months. None of the varieties tested were immune to CBSV or UCBSV. Mixed infection of both UCBSV and CBSV was evident in all cassava genotypes. Genotypes varied in symptom expression and relative virus load of UCBSV and CBSV which also varied over time, indicating differential genotype response to virus infection.

### Shoot symptoms

In accordance with previous work [5] considerable variation was observed in incidence and severity of shoot symptoms. No shoot symptoms were observed in Kiroba, NASE 1, NASE14 and NASE19 yet 100% incidence was observed in Albert and TME 204 which also showed mean severities of 3 and 4.07 respectively. In many cases there was a positive relationship between shoot incidence and severity and root necrosis incidence and severity. A few exceptions included genotype NASE 19 which had no shoot symptoms, but root necrosis incidence of 67% with a maximum of 5. Reasons for this disparity remain unclear although [24] reported the possibility of localization of the virus in the base of the plant.

To date the focus in breeding has been on reducing roots necrosis, and the expression of shoot symptoms has been considered acceptable if root symptoms are absent, infrequent or very mild [25]. However [26] indicated that yield reductions resulting from shoot symptoms could be larger than losses due to root necrosis. This suggests that future cassava breeding should incorporate selection for reduced shoot incidence and symptoms [25].

### Root necrosis

Variation in root necrosis was observed as expected and was consistent with previous observations of CBSD [5]. Namikonga, NASE1 and Kiroba had an incidence of root necrosis less than 20%, and maximum severity scores of 2, 2 and 3 respectively. AR40-6 had an incidence of 30.3%, but a mean severity score of 1.09 and a maximum of 3. It is likely that these genotypes possess elements that will be useful in a CBSD resistance breeding, but these must be considered in relation to virus load. In three different studies Namikonga, also known as Kaleso, showed the highest general combining ability for resistance to CBSD [27–29]. This cultivar is now widely used by national breeding programs in the region.

Interestingly NASE14 remained asymptomatic for CBSD for both shoot and roots while the few that succumbed to infection showed very high root severity (4 or 5) and incidence (90 -100%). This was coupled with reduction in growth and in some cases dieback. This response will have to be confirmed through fingerprinting of individual plants to ensure uniformity in genotype and diagnostics on individual plants to dismiss the possibility of ‘escapes’. It can be hypothesized that there could be a threshold at which the virus overcomes the plant defense mechanism thereby causing necrosis. This hypothesis should be further investigated in ‘degeneration’ trials. Such studies will be important in determining resistance durability and in designing seed systems for cassava planting materials. Similarly derivatives of *M. melanobasis* (now regarded as *M. esculenta* subsp. *flabellifolia* [16]), were observed to be highly resistant and rarely became diseased but, when present, the symptoms were severe [18]. This was attributed to a low capacity to recover from symptoms with new symptom-free growth.

### Detection and quantification of (U)CBSV

The large differences in virus load of UCBSV (low) and CBSV (high) in TZ/130, AR40-6 and NASE 1 could be due to competition among the viruses with CBSV outcompeting UCBSV, differences in pathogenicity or differential reaction of genotype to each virus. Higher virus loads of epidemic CBSV than endemic UCBSV in cassava varieties and herbaceous hosts have been observed previously [30], greater transmission rates [30,21] and more severe symptoms [8,30]. Due to lack of information regarding interaction of the viruses and their relative competitiveness, the two virus species were both considered together here, and no inferences made on whether a genotype was resistant or susceptible to either virus.

Relative virus loads changed through the growing season with NASE 14 and Namikonga showing a decline in relative UCBSV and CBSV loads at 7MAP and Kiroba at 7MAP and 9MAP respectively. Kiroba had tested positive for UCBSV at 5 and 7MAP but this could not be detected at 9 and 11MAP (Table 3, Figure 3). A similar situation was observed in Kaleso (equivalent to Namikonga) and Kiroba in the middle of an infection time course experiment [9]. Declines were also observed in AR40-6 (UCBSV) and NASE 1 (CBSV). This phenomenon indicates either competition among viruses (eg. AR40-6) and/or activation of an antiviral defense system, which could include RNA interference [31]. The fact that this mechanism allows the virus to accumulate in the plant for some time before it is reduced means that this mechanism is not constitutive, but inducible. Recovery has been observed during periods of rapid growth (9 to 15MAP) [32,18] but it is yet to be determined whether this recovery coincides with reduction in virus load. In addition, it would be interesting to observe the dynamics of virus load if infected cuttings were used, or in ‘degeneration’ trials, as observations may be specific to newly infected cuttings.

### Correlation of virus load with symptom expression

Symptom expression has been shown to correlate with virus load in different organs of two genotypes [22] although large standard deviations at high CBSV levels were also observed. For genotypes that showed shoot symptoms, symptom expression was highly correlated with at least one of the viruses (either UCBSV or CBSV) with the exception of TZ/130 which had mild shoot symptoms (maximum score 2), but very high relative CBSV load. Thus it appears that a correlation between virus load and symptom expression holds true for at least one virus species in susceptible genotypes, but breaks down in genotypes showing some resistance or tolerance. Regarding relative virus load and root necrosis, there were a number of exceptions where the correlation did not hold true, and which define the ‘categories’ outlined below. NASE 1, TZ/130 and to some extent AR40-6 appeared to allow accumulation of virus while restricting symptom expression. It is important that such genotypes are not distributed as varieties directly as they would serve as inoculum reservoirs and accelerate virus spread. They could be crossed with varieties that are able to restrict virus accumulation to combine this trait with reduced symptom expression. NASE 14 and NASE 19 on the other hand appear to keep virus load low, but express a severity of root necrosis up to Class 5 with relatively high incidence. This apparent break in correlation indicates distinct resistance mechanisms that govern symptom expression and virus accumulation.

### Categories of disease response

Virus resistance terminology is a contentious issue on which there is no general agreement and a number of definitions exist [33,34]. According to [33] truly resistant cultivars are not readily infected, even when exposed to large amounts of vector-borne inoculum and when infected they develop inconspicuous symptoms that are not associated with obvious deleterious effects on growth and yield and support low virus content and thus to be a poor source of inoculum. The term ‘resistance’ is therefore a combination of two different components: virus titre or load and symptom expression.

CBSD shoot and root necrosis incidence and severity and relative virus load suggest that at least two main mechanisms may be operating, one that seems to restrict symptom expression under high virus load, and the other that seems to inhibit virus accumulation. The ability of some varieties to impair the replication of CBSVs has been observed in cassava [21,23], although documented cases of this in other plant species are rare [35,36]. Various genotypes seem to possess none, either one, or a combination of these mechanisms. Based on this, four categories of genotypes were recognized according to response to the CBSD viruses:

1. Namikonga showed resistance to field disease symptoms and kept virus loads low relative to the susceptible genotypes. Namikonga remained symptomless apart from one plant that showed root necrosis with maximum score of 2 (very minor discoloration). Relative virus load declined from 7 to 9 and 11MAP for both UCBSV and CBSV respectively in Namikonga. This indicates an ability to restrict virus accumulation and resist root necrosis development. Based on relative virus load, under glasshouse conditions with graft inoculation ‘Namikonga’ has been classified as ‘resistant’ [21] and our results concur with this.
2. This category comprises genotypes that appear to keep virus loads low, but express a range of symptoms from slightly more severe, at a slightly higher incidence, than Category 1 (Kiroba) to those that show root necrosis up to Class 5. Kiroba had an average root necrosis of 1.07, maximum score 3 and an incidence of 14.3%, but kept virus loads low. A decline in virus loads was observed from 7MAP for UCBSV, and dramatically from 9MAP for CBSV. Kiroba has previously been classified as ‘tolerant’ due to intermediate virus loads [21]. Here Kiroba has an intermediate position between Categories 1 and 2 but is placed in Category 2 because of a maximum root necrosis score of 3. NASE 14 and NASE 19 are included in this category as they kept virus loads low and showed no shoot symptoms but showed root necrosis up to maximum score 5. NASE 14 showed a decline in CBSV and UCBSV relative virus load from 7MAP, whereas in NASE 19 this decline occurred from 9MAP for CBSV and relative virus loads continue to rise for UCBSV albeit at extremely low levels. No consistent relationship between relative virus load and symptom expression was observed in NASE 14 and NASE 19, although this may have been obscured by pooling leaf samples prior to real-time RT-PCR.
3. This category comprises genotypes that harbor high virus loads but show relatively mild symptoms with low incidence. NASE 1 showed mild symptoms with no shoot symptoms, a maximum root necrosis of 3 with 73.3% of plants remaining symptom free. Similarly TZ/130 showed mild symptoms with 17% shoot symptoms and a maximum score of 2, and a mean root necrosis score of 1.2, and a maximum of 4. AR40-6 could also be considered in this category with maximum root necrosis of score 3, and a mean of 1.09, although it did show a high level of shoot symptom incidence (52%) and a maximum score of 3. NDL06/132 also had a high incidence of shoot symptoms (67%), but moderate root symptoms (minimum 2, maximum 3). The four varieties did harbor high levels of CBSV and thus seemed to be able to restrict symptom expression to some extent but not CBSV load. NDL06/132 also had a high UCBSV load. This again brings into question the relationship between symptom expression and relative virus load observed by [20,21,23].
4. Kibaha, Albert and TME 204 were susceptible both in terms of field symptoms (both shoot and root necrosis) and virus load, having high relative virus loads for both UCBSV and CBSV.

Relating these four categories to conventional terminology, Category 1 can be equated to ‘resistance’, Category 2 can be considered ‘tolerant (restricted virus load)’, Category 3 ‘tolerant (restricted symptom incidence and severity)’ and Category 4 as ‘susceptible’. It is envisioned that classifying genotypes in this way will not only make biological sense to ‘field breeders’, but, by providing transparency in terms of symptoms and virus load, will help breeders in making choices of parents for crossing. For example, it may be prudent to cross a variety showing resistance to symptom expression with one showing restricted virus accumulation. It is worth noting that only leaf samples were used for analysis of virus accumulation. Therefore it is possible that those genotypes that show reduced root necrosis (Kiroba, Namikonga and NASE 1) allow virus accumulation in the leaves but restrict the translocation of the virus to the roots. This requires further investigation. In addition, samples were pooled across plants, which obscures among plant variation.

### Implications for cassava breeding

The above results indicate at least two possible mechanisms of resistance/tolerance to CBSVs. This is consistent with earlier findings. Namikonga and possibly Kiroba are direct derivatives of the Amani breeding program, whereas NASE 14 and NDL06/132 have Amani breeding germplasm in their pedigrees (Table 5). The Amani breeding program involved crosses with wild species, followed by up to three back-cross generations and inter-crossing of backcross selections. The low harvest index of Namikonga is likely to be due to residual non-storage root producing wild species genome.

**Table 5:** **Pedigree information of varieties included in this study**

**Variety**	**Pedigree**	**Possible source of CBSD resistance/tolerance**
Namikonga	Known as ‘Kaleso’ in Kenya. Third backcross from inter-specific hybrid (46106/27) from *M. glaziovii* from Amani breeding program [29,5]	*M. glaziovii*
NASE 1	Introduced as TMS 60142 from IITA in early 1980s	Unknown
AR40-6	Bred by CIAT. Has 12.5% from wild species *M. esculenta* subsp. *flavellifolia* and 50% from CMD resistant variety C39.	
Kiroba	Landrace from Tanzania	Unknown
TZ/130	Selection made in Uganda from open pollinated seeds introduced from Tanzania	Unknown
NASE 14	Also known as MM96/4271. Bred by IITA.	
NASE 19	Also known as 72 TME 14. It is a half-sib of TME 14, a landrace from West Africa introduced by IITA	Unknown
NDL06/132	Breeding line selected at ARI Naliendele in southern Tanzania. It is an S1 self of variety NAL 90/34 which showed strong resistance to CBSD [5] and is half sib of Kibaha. which has *M. e.* subsp. *flabellifolia* background.	
Albert	Local landrace from Tanzania	Susceptible check
Kibaha	*M. e.* subsp. *flabellifolia* background.	
TME 204	Introduction from IITA.	Susceptible check

The breeding strategy was likely to have resulted in the combination of resistance genes from several sources [5]. Inter-crossing among them would concentrate resistance genes and allow recessive genes to be expressed [5]. CBSD resistance was observed to be satisfactory in the backcrosses and was maintained in the inter-crosses [18]. This pool of resistance factors may also have been augmented by local cultivars that were unintentionally selected in areas of high disease pressure for resistance/tolerance to CBSD. Similarly [5] concluded that the type of ‘resistance’ expressed seems to differ between cultivars. They observed variations in symptoms as observed in this study.

CBSD resistance has been reported to be quantitative and recessive with both additive and non-additive genetic effects [29,32]. However, the additive effects were more important, implying that intra-population selection methods should be effective in accumulating favorable alleles in breeding materials [37]. In addition, resistance to CBSD and CMD were inherited independently of each other and showed continuous variation in their expression.

This was a preliminary study to investigate virus load in genotypes with contrasting symptoms under field conditions. It was based on responses in a single growing season (12 months) and thus broadening our understanding on the concept of virus resistance (viral load) and disease resistance (symptom expression). It is important that disease observations and virus load are measured over several years and across a broader range of environments. Studies to identify quantitative trait loci are underway to further extrapolate resistance mechanisms as are differential gene expression studies based on RNASeq [21] (Ferguson *personal communication*).

## Conclusion

This study reveals a complex situation with regard to resistance or tolerance to CBSD. The genotypes not only showed variation in shoot and root necrosis incidence and severity, but also relative virus load of UCBSV and CBSV, and with time. Substantially different levels of the virus species were found in many genotypes suggesting either resistance to one virus species or the other, or some form of interaction, antagonism or competition between virus species. It appears that virus load is not always correlated with symptom expression, so some genotypes are able to withstand high levels of virus while showing mild symptoms (NASE 1, TZ/130, AR40-6 and NDL06/132). Other genotypes are able to restrict virus accumulation or have a system of recovery (Kiroba, NASE 14, NASE 19). Some genotypes may possess a combination of these different mechanisms (Namikonga). Historical evidence from the Amani breeding program, based on backcrossing from inter-specific crosses and inter-crossing of inter-specific derivatives supports the hypothesis and evidence for different mechanisms of resistance including those that restrict virus accumulation and those that restrict symptom expression. A substantial amount of research still needs to be undertaken to fully understand the bases of resistance. This information will be useful to plant breeders in informing breeding strategies and restricting virus spread. For durable resistance, various mechanism can be combined or exploited by considering both virus and disease resistance in different genotypes.

## Methods

### Selection and field establishment of cassava genotypes

Eleven cassava genotypes selected from Uganda and Tanzania were screened for field resistance to both UCBSV and CBSV in Uganda. Tanzanian genotypes reported to be resistant/tolerant in Tanzania were AR40-6, NDL06/132, Kiroba and Namikonga (also known as Kaleso), and Ugandan genotypes reported to be tolerant in Uganda were NASE 14 (MM96/4271), 72-TME 14 (NASE 19), NASE 1 and TZ/130 (Table 5). Genotypes Albert from Tanzania, and Kibaha and TME 204 from Uganda were included as susceptible controls. Genotypes from Tanzania were obtained as virus-free tissue culture plantlets while those from Uganda were sourced as stakes from CBSD disease-free areas. All planting material was diagnosed as free of (U)CBSV prior to planting. Tissue culture plantlets were hardened according to [38]. Field trials were established in the first rains (March – May) of 2012 at National Crops Resources Research Institute (NaCRRI), Central Uganda (lat/lng: 0.529, 32.612, Alt 1222 m), an area with high CBSD and whitefly pressure [39]. Test genotypes were established in two row unreplicated plots each containing 10 plants with a spacing of 1 m × 1 m. Each plot was separated by a CBSV/UCBSV infected spreader row of TME 204. Plants of TME 204 used in the spreader rows were obtained in fields that had a CBSD incidence of 100% and a mean severity of 4 and 4.5 for shoot and root necrosis respectively. This selection was done to ensure that infector line had high viral load to effectively augment CBSD pressure. The genotypes were grown for 12 months under rainfed conditions on a sandy-loam soil and no fertilizer or herbicide was applied. Regular weeding was undertaken.

### Field evaluation

The trial was monitored for above ground symptoms during the crop growth period and symptoms in the roots after harvest. Symptoms on shoots (leaves and stems) were recorded on each plant at three, five, seven and nine months after planting (MAP). A severity score of 1–5 [39] was adopted where 1- no apparent symptoms, 2- slight foliar chlorotic leaf mottle, no stem lesions, 3- foliar chlorotic leaf mottle and blotches with mild stem lesions, no dieback, 4- foliar chlorotic leaf mottle and blotches and pronounced stem lesions with no dieback and 5- defoliation with stem lesions and pronounced dieback. A mean shoot severity score was then calculated per genotype based on all individual plant scores per genotype at 9 MAP.

Severity scores for root necrosis were also taken on all roots harvested per plant at 12MAP. At harvest, each root was cut across into slices approximately 5 cm apart, and the maximum severity score taken for each root where 1- no necrosis, 2- mild necrotic lesions (1-10%), 3-pronounced necrotic lesions (11-25%), 4-severe necrotic lesions (26-50%) and 5- very severe necrotic lesions (>50%). A root disease severity mean value was calculated on a per plant basis, and then averaged over plants to give a mean value for each genotype. Per plant mean root necrosis incidence was quantified as a ratio of the number of roots showing root symptoms to the total number of roots harvested per plant. This was averaged to give a value per genotype.

In addition, at 12 MAP fresh shoot biomass (stems and leaves) and roots per plant were weighed separately and harvest index calculated on a plot basis as the ratio of storage root weight to total plant biomass and storage root weight [40]. This was used as an indirect assessment of fresh root yield.

### Sample collection and RNA extraction

At 3MAP, six plants per genotype that showed leaf symptoms were tagged for leaf sampling, whereas sampling of six plants of symptomless genotypes was done through random selection. At 3,5,7,9 and 11 MAP a mature leaf (second level from the bottom) was sampled from each tagged plant and stored at −84°C. At the beginning of the trial, many of these genotypes did not show foliar symptoms for the first 3 MAP. Leaves were therefore pooled together to avoid or reduce false negative probability for detection and quantification of CBSV/UCBSV in cassava tissues [41] and also to reduce the cost of analysis. Approximately 100 mg of leaf tissue was ground into fine powder using liquid nitrogen and a small hand roller. To this was added 1 ml CTAB grinding buffer containing 2% CTAB, 100 mM Tris – HCl, pH 8.0, 20 mM EDTA and 1.4 M NaCl. This was then incubated at 65°C for 15 minutes after which 700 μl of chloroform: isoamyl alcohol (24:1) was added and centrifuged at maximum speed in a microfuge for 10 min at room temperature. The aqueous layer that formed was removed and transferred into a clean nucleases free 1.5 ml microfuge tube after which an equal volume of 4 M LiCl was added and incubated overnight. The mixture was centrifuged for 30 min at maximum speed of 13,000 g at 4°C to pellet the nucleic acids.

The pellet was re-suspended in 200 μl of TE buffer containing 1% SDS after which 100 μl of 5 M NaCl and 300 μl of ice cold iso-propanol was added and the mixture incubated at −20°C for 30 min. After incubation, the mixture was centrifuged for 10 min at 13,000 g to pellet the nucleic acid. The pellet was then washed by adding 500 μl of 70% ethanol and centrifuged for 4 min at 4°C. The ethanol was decanted off and the pellet dried and re-suspended in 50 μl of nuclease –free sterile water. RNA quality and quantity was measured using a Nanodrop ND-1000. Due to differences in RNA quantity, the samples were normalized to a working concentration of 100 ngμl^−1^ by addition of an appropriate amount of sterile water.

### Quantitative real time PCR for CBSV and UCBSV

The RT-PCR assay used was based on TaqMan chemistry using primer and probe sequences reported by [41] except that the CBSV probe was 5’-FAM-TAMRA-3’ labeled and the UCBSV probe was 5’-VIC-TAMRA-3’ labeled. In addition, COX (cytochrome oxidase) was used as an internal control with primers COX-F (5’- CGTCGCATTCCAGATTATCCA-3’), COX-R (5’- CAACTACGGATATATAAGRRCCRRAACTG-3’) and probe (5’- [FAM]-AGGGCATTCCATCCAGCGTAAGCA-[TAMRA]-3)’. COX is a widely used housekeeping gene to normalize cycle threshold (Ct) values and was validated by [41] for use with CBSV and UCBSV quantification using real-time PCR. For each RNA sample, two technical replicate reactions were prepared containing 12.5 μl of Maxima Probe qPCR Master Mix (2X) (Fermentas), 7.5 μM of each forward and reverse primer, 5 μM Taqman probe, 100 ng of template, MMLV-Reverse transcriptase and nuclease free sterile water to volume of 25 μl. In addition, non-template water control was included on every plate. The reactions were incubated for 60 min at 42°C then initial denaturation step run for 10 min at 95°C followed by 40 cycles of denaturation for 15 sec at 95°C, annealing for 30 sec at 60°C and extension for 30 sec at 72°C.

All real-time PCR reactions were performed on an Applied Biosystems’ One Step Plus® sequence detection system (Applied Biosystems). The generated cycle threshold (Ct) values were used to determine the fold change in expression of a target gene relative to that at 3MAP for both CBSV and UCBSV using a comparative 2^-∆∆Ct^ method as described by [42] where ∆∆Ct = (Ct_target_-Ct_Cox_)_time x_ – (Ct_target_-Ct_Cox_)_3 months_ and where x is time (5, 7, 9, 11 MAP). All genotypes that had C_t_ value of 40 for UCBSV or CBSV were considered to be free of these viruses. The fold changes were transformed to log10 and plotted against time (MAP) to monitor the relative accumulation of virus in different genotypes with time. In addition log10 fold changes were regressed against mean shoot symptom scores at 3,5,7,9 and 11MAP and the coefficient of determination (r^2^) calculated.

## Additional file

Additional file 1: Table S1. Absolute Ct values of technical replicates for UCBSV in selected genotypes in Uganda observed at 3,5,7,9 and 11 MAP. **Table S2.** Absolute Ct values of technical replicates for CBSV in selected genotypes in Uganda observed at 3,5,7,9 and 11 MAP.
