# Supplementary material for: Field evaluation of selected cassava genotypes for cassava brown streak disease based on symptom expression and virus load
Source: Virol J. 2014 Dec 20;11:216. doi: 10.1186/s12985-014-0216-x (PMC4304613; doi:10.1186/s12985-014-0216-x)
Supplement: Additional file 1: Table S1. — Absolute Ct values of technical replicates for UCBSV in selected genotypes in Uganda observed at 3,5,7,9 and 11 MAP. Table S2. Absolute Ct values of technical replicates for CBSV in selected genotypes in Uganda observed at 3,5,7,9 and 11 MAP. [file 12985_2014_216_MOESM1_ESM.pdf]

Table S1: Absolute C_t values of technical replicates for UCBSV in selected genotypes in Uganda observed at 3,5,7,9 and 11 MAP

	Ct value of UCBSV									
Genotype	3 MAP		5 MAP		7 MAP		9 MAP		11 MAP	
	Rep 1	Rep 2	Rep 1	Rep 2	Rep 1	Rep 2	Rep 1	Rep 2	Rep 1	Rep 2
NASE 14	37.24	37.00	35.67	36.25	31.23	31.63	35.23	36.47	37.12	37.62
AR40-6	28.13	30.65	27.69	26.69	30.75	16.43	20.88	20.72	34.91	35.11
Kibaha	40	40	35.82	35.26	34.06	32.74	30.52	31.00	27.31	27.37
NDL06-132	37.29	38.97	34.91	33.13	32.57	32.37	25.74	25.62	19.61	19.51
Kiroba	40	40	34.74	33.78	34.43	34.10	40	40	40	40
Albert	33.61	34.73	32.61	33.19	31.97	31.47	20.65	20.25	17.28	17.04
NASE 1	40	40	40	40	40	40	39.06	38.91	33.15	33.33
Namikonga	38.47	38.07	37.22	37.90	37.62	34.84	36.01	37.89	35.68	36.07
Tz/130	35.19	37.49	39.51	38.53	34.91	37.17	36.21	37.31	36.35	35.84
NASE 19	37.08	37.43	37.92	38.66	33.84	35.88	35.01	34.07	33.99	34.33
TME 204	39.72	39.18	27.01	28.27	20.43	21.85	18.52	19.56	18.52	18.42

Table S2: Absolute C_t values of technical replicates for CBSV in selected genotypes in Uganda observed at 3,5,7,9 and 11 MAP

Genotype	Ct value of CBSV									
	3 MAP		5 MAP		7 MAP		9 MAP		11 MAP	
	Rep 1	Rep 2	Rep 1	Rep 2	Rep 1	Rep 2	Rep 1	Rep 2	Rep 1	Rep 2
NASE 14	21.32	23.86	19.67	19.41	16.31	16.37	29.49	29.14	33.53	33.22
AR40-6	38.91	38.41	36.91	35.83	31.54	32.99	31.74	31.17	20.87	20.89
Kibaha	40	40	26.72	27.20	23.71	23.71	20.79	20.80	24.82	25.31
NDL06-132	35.50	35.77	21.55	20.11	14.79	15.27	32.83	34.77	17.49	17.16
Kiroba	40	40	35.44	36.00	31.00	31.09	31.49	30.93	34.32	34.84
Albert	36.15	35.96	21.34	21.94	18.49	18.69	19.99	19.86	19.60	19.61
NASE 1	33.88	36.12	27.88	27.24	16.27	16.25	20.84	20.88	18.16	18.39
Namikonga	40	40	26.66	27.54	22.47	22.84	30.54	30.34	21.37	21.17
Tz/130	37.98	37.72	23.47	23.15	19.59	19.68	31.07	30.39	21.26	21.28
NASE 19	40	40	33.69	32.33	31.09	30.86	29.66	29.64	33.20	33.88
TME 204	36.11	35.07	19.94	18.90	14.87	16.17	18.00	18.66	16.59	17.99
